# Why is tractable vision loss in older people being missed? Qualitative study

**DOI:** 10.1186/1471-2296-14-99

**Published:** 2013-07-16

**Authors:** Kalpa Kharicha, Steve Iliffe, Sybil Myerson

**Affiliations:** 1 Research Department of Primary Care and Population Health, UCL, Royal Free Campus, Rowland Hill Street, London NW3 2PF, UK

**Keywords:** Older people, Vision loss, Case finding

## Abstract

**Background:**

There is compelling evidence that there is substantial undetected vision loss amongst older people. Early recognition of undetected vision loss and timely referral for treatment might be possible within general practice, but methods of identifying those with unrecognised vision loss and persuading them to take up services that will potentially improve their eyesight and quality of life are not well understood. Population screening does not lead to improved vision in the older population. The aim of this study is to understand why older people with vision loss respond (or not) to their deteriorating eyesight.

**Methods:**

Focus groups and interviews were carried out with 76 people aged 65 and over from one general practice in London who had taken part in an earlier study of health risk appraisal. An analytic induction approach was used to analyse the data.

**Results:**

Three polarised themes emerged from the groups and interviews. 1) The capacity of individuals to take decisions and act on them effectively *versus* a collection of factors which acted as obstacles to older people taking care of their eyesight. 2) The belief that prevention is better than cure *versus* the view that deteriorating vision is an inevitable part of old age. 3) The incongruence between the professionalism and personalised approach of opticians and the commercialisation of their services.

**Conclusions:**

The reasons why older people may not seek help for deteriorating vision can be explained in a model in which psychological attributes, costs to the individual and judgments about normal ageing interact. Understanding this model may help clinical decision making and health promotion efforts.

## Background

There is compelling evidence that there is substantial undetected vision loss amongst older people [1]_._ By the age of 65, 1 in 6 will become blind or partially sighted [2], and every day around 100 people in the UK start to lose their sight [3]. Between 12 and 50% of older people have undetected vision loss due to refraction changes, cataract, glaucoma and retinal disease, with higher prevalence amongst women and risk increasing rapidly with age [1,4].

The impact of vision loss on quality of life, activities of daily living and accidents (including falls), is also well documented, adding further weight to the argument for focusing on prevention, early detection and timely access to treatment in this age group. The UK Vision Strategy [5], launched in April 2008, aims to improve the eye health of the nation by eliminating avoidable sight loss, delivering excellent support to those with visual impairment and enhancing the inclusion, participation and independence of blind and partially sighted people.

It is not yet clear why remediable eye disease is being missed in an advanced primary care system with easily accessible doctors and nurses who can administer simple screening tests, and a widespread network of community opticians who offer free screening to older people. The evidence for community based screening for asymptomatic visual impairment from the most recent update of the Cochrane review, shows that such screening does not lead to improved visual function in the older population [6].

From the accompanying study [7] we know that limited education is an independent determinant of not having eye tests, as well as a factor associated with vision loss. There are other factors, not identified in this study, which determine uptake of eye testing. Further exploration is needed to identify these factors and lead towards efficient case finding.

Although some qualitative work has been performed with older people with vision loss, this has either focused on a particular group like those receiving social care [8], on a specific need like housing [9] or on communication strategies [10]. An open-ended study that seeks to understand why older people with vision loss respond (or not) to their deteriorating eyesight could be helpful. This paper describes such a study.

## Methods

### Recruiting older people

**Figure 1:**
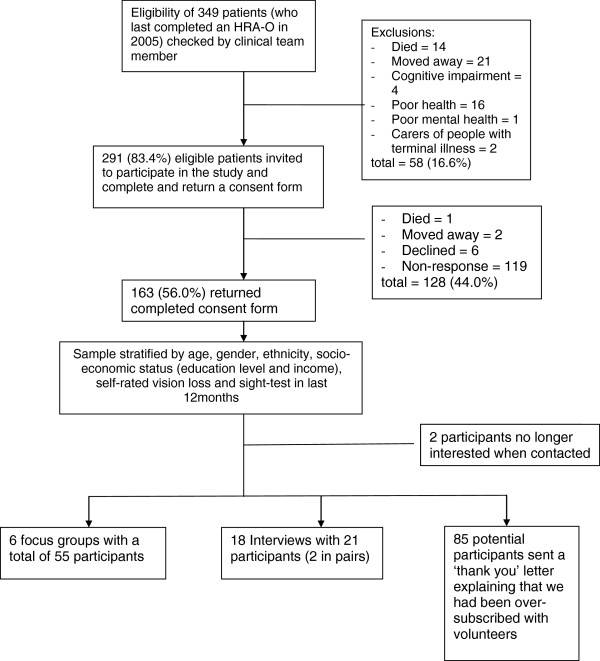
Recruitment and sampling of older people for interviews and focus groups.

Three primary care teams involved in earlier research into health risk appraisal in older people (conducted between 2001 and 2005) were invited to participate in a qualitative study of reasons why remediable vision loss is being missed. One large group practice located in suburban London, agreed to join the study. The primary care team was asked to check whether patients who had completed a health risk appraisal questionnaire (which included the National Eye Institute Visual Function Questionnaire (NEI-VFQ) [11-14]) in 2005 remained eligible to participate. That is, they were still registered at the practice and had no new diagnoses of major physical, mental, cognitive or terminal illness. This group was posted an invitation to join the study with an information sheet, asked to complete a consent form and to indicate their preference for participation in either a focus group at the practice or face-to-face interview, either at the practice at their home. A focus group method was chosen because of this method’s value in exploring people’s knowledge and experience, and the reasons underpinning their beliefs and attitudes [15]. Individual interviews were offered as well as group discussion to gain insider perspectives of issues that could be potentially distressing as well as detailed understandings of how local services functioned, and frames of reference for visual function loss [16]. A choice of participation in a focus group or in an interview was offered to include older people who might not feel comfortable in a group setting, or who might find attendance at a group difficult because of limited mobility or access to transport. To ensure generalisability of the conceptual analysis the responding sample was stratified by age, sex, ethnicity, socio-economic status, self-rated vision loss and recent vision testing to ensure a range of individuals and experiences were represented. The derivation of the sample is shown in Figure 1.

A semi-structured interview schedule, informed by existing literature, was developed with input from the study’s Advisory Group and piloted; this is shown in Table 1. The first three questions shown in Table 1 were used to initiate and promote discussion in the focus groups. Topics included experiences of changes in vision, how people knew when to get their eyesight checked, why some people tolerate worsening eyesight more than others, whether sight loss was considered an unavoidable part of getting older, experiences of visiting opticians and discussions with primary care about their eye health. The pilot did not change the content of the schedules and this data was included in the main sample in terms of analysis.

**Table 1:** Questions and prompts used in interviews (the first three questions were used in focus groups)

**Questions**	**Prompts**
*Eyesight tends to get worse as we get older*. *How do people know when to get their eyesight checked*?	Explore experiences of visiting opticians –
*Why do some people put up with worsening eyesight more than others*?	• what made you decide to go to the optician? (reminder letter?)
*Do you think sight loss is an unavoidable part of getting older*?	• how often do you tend to see an optician?
*Have you ever spoken to your GP about any problems you have with your sight*?	• How often do you think you should see an optician? (if discrepancy with response to above, ask why this is)
	• how easy is it to see an optician (finding an optician/booking an appt/access)
	• how useful was the appointment?
	• what was the result?
	• did you follow their advice? –if not, why not?
	• were you referred to your GP or the hospital?
	• were you offered any special spectacles or magnifiers?
	• was there any financial impact? (NHS help?)
	Explore as above:
	• what made you decide to go to talk to your GP?
	• how useful was the discussion?,
	• what was the outcome?,
	• did you follow their advice? –if not, why not?

NHS Research Ethics Committee approval for the study was given by Harrow Research Ethics Committee.

### Obtaining data

Focus groups were led by a facilitator who was a qualitative researcher experienced in both interviewing and group facilitation, with an observer (with a background in qualitative medical sociology) taking detailed notes. The facilitator directed the discussion to consider a number of questions, and focused attention on achieving a common understanding of these questions and their answers [17].

All interviews and focus groups were audio recorded and transcribed verbatim and were continued until no new themes emerged from the discussions.

### Analytic methods

The approach taken allowed for the public readjustment of definitions, concepts, and hypotheses [18]. In this study, a modified analytic induction technique based on the accounts described by Johnson [19] and Bondas [20] was used to compare informants’ accounts so as to identify similarities and differences, thereby constructing the uniformities underlying and defining the emergent themes/categories. Analytic induction (AI) is defined as the intensive examination of a strategically selected number of cases so as to empirically establish the causes of a specific phenomenon. Shared features from the group or individual accounts were used to generate themes/categories. ‘Deviant’ cases were re-examined to ascertain if re-categorisation was needed to incorporate them.

All transcripts were read independently by three researchers, who met to compare themes/categories and differences in opinion discussed until resolved. The themes were presented to a multi-disciplinary Advisory group including older people with vision loss who had not participated in the interviews or nominal groups, for further discussion, and tapes were given to members of the Harrow Association for the Blind to review their contents and compare with the themes.

### Synthesis of findings

The findings were then presented to an expert panel including older people and representatives of the Thomas Pocklington Trust, alongside evidence from the existing literature, to try and achieve a consensus on the factors that impede patient and professional action on vision loss. With the expert panel, we developed a model that describes 1) ‘patient barriers’ to the take up of remedial vision services (i.e. the factors that dissuade individuals from seeking professional help with vision problems, or acting on professional recommendations), that is presented in this paper and 2) how general practitioners, practice nurses and community opticians understand patients’ reasoning about the significance and tractability of visual loss, and to identify ‘professional barriers’ to improving visual function in later life.

The trustworthiness of the data and our analysis of it [21] was framed in terms of its credibility to others with experience of the topic, transferability to other settings, dependability (depth of description of methods, peer analysis of data, third party evaluation of data gathering) and confirmability (by independent review of the data). Confirmability was sought using the two phase process described above, and the credibility, transferability and dependability were tested by presenting our findings to the project’s multidisciplinary Advisory group.

## Results

We held six focus groups, each scheduled for no more than 1.5 hours, with 55 older people who had completed the questions on visual function in 2005. There were also eighteen individual interviews with 21 older people from the same group who did not wish to attend a focus group; two of these interviews were with a pair of participants, at their request. Interviews lasted between 25 and 70 minutes.

### Findings from interviews and focus groups with older people

The focus groups of older people were a rich source of ideas, and provided all the themes reported here. They debated three themes, each of which had a dichotomous content. The themes were:

1. The capacity of individuals to take decisions and act on them effectively *versus* a collection of factors which acted as obstacles to older people taking care of their eyesight.

2. The belief that prevention is better than cure *versus* the view that deteriorating vision is an inevitable part of old age.

3. The incongruence between the professionalism and personalised approach of opticians and the commercialisation of their services.

The interviews, whilst producing some interesting examples and quotes, only validated these themes, and added no new ones. The interview data nevertheless contributed to the model development.

### Theme 1. Deciding when to seek help vs. Obstacles to help

Decisions about getting eye tests done or treatment started seemed to hinge on significant events, as shown in quotes 1 to 4 in Table 2 Other important changes that were mentioned were difficulty in watching television, driving (especially at night), and difficulty in reading bus numbers. Decision-making judgements about the frequency of follow-up needed are illustrated by quotes 5 and 6 in Table 2.

**Table 2:** Deciding when to seek help

	
1	“*I made the decision*, *when I was about to cross the road*, *there was a car coming and I could not see it properly*, *I had the operation then*”. [*FG1*]
2	“*I have to get* (*my glasses changed*) *when my arm gets too short*”. [*FG2*]
3	“*Obviously when you are reading* (*and*) *the words start to swim around*….*you just know you have got to go and get your eyes tested*”. [*FG2*]
4	“*Probably I couldn*’*t see the small print of a bible I was reading*. *Had to get larger print*. *So that was probably it*”. [*FG2*]
5	“*I*’*ve had the same prescription now for probably about ten years*…(*If I had not lost my glasses*) *I probably would not have gone* (*to the optician*) *because*…*there appeared to be no change*, *I could still read at the same distance*, *the same size of print*…” [*FG6*]
6	“*You probably realise that you*’*re getting* (*cataract*) *or something and I believe there is no great hurry to get it done*, *they make you wait a bit anyway*. *So I may not go every couple of years*.” (*laughs*) [*FG6*]

The main factors which explain the failure of older people to seek investigation or treatment for deteriorating eyesight were seen as denial, fear and costs. Denial was seen as a feature of not accepting ageing or limitations in activity, which resulted in worsening vision being described as normal (quotes 1 to 3 in Table 3). Fear could be part of denial but was also a feeling that everyone had, to some extent, before seeking investigation or treatment (quotes 4 to 6 in Table 3). This fear could be specific (about what might be found) or general (about professionals and hospitals). The cost of buying and updating lenses and glasses was raised by most participants, despite the availability of free eye examinations for this age group (quotes 7 and 8 in Table 3). However, costs were seen as only a partial barrier (by this self-selected group), operating to reinforce other themes that inhibited help-seeking.

**Table 3:** **Denial**, **fear and costs**

	
**Denial**	1. “*A lot of people have the attitude that* ‘*there*’*s nothing wrong with my sight*, *the print is getting smaller*!” [*FG2*]
	*2*. “*I thought I would just leave it*, *leave it*, *you know*. *Keep leaving it and see what happens*….” [*FG3*]
	*3*. “*There*’*s many people who do not want to know*”
	“*I come from a family of people who don*’*t want to know*”
	“*That*’*s actually silly*”
	“*I know it is*. *I can*’*t convince them*”. [*FG5*]
**Fear**	4. “*I am dead scared of even having my cataracts done*…*because my husband lost his sight*. *That scares me*.[*FG3*]
	5. “..*it*’*s a little bit of the fear of the unknown*, *because*…*the idea that somebody is messing about with your eyes*, *it*’*s really frightening*”. [*FG3*]
	*6*. “*I think it*’*s sometimes fear of going blind that makes one go and check on your eyes*. *My mother and grandfather went blind*, *so it does make you a bit nervous*”.
	“*And that works the other way sometimes with some people*. *They don*’*t want to go*”. [*FG5*]
**Costs**	*7*. “*you made an interesting statement* … *that the services were free*. *It cost me* £*300 the last time I was there*!” [*FG1*]
	*8*. “*Costs so much I go alternate years*”

The groups had views on the sorts of people who would not seek investigation or treatment for worsening vision (quotes 1 to 3 in Table 4). They were described as more likely to be men, living alone, perhaps with reduced mobility. How these factors interacted when combined, however, was unclear from the discussions, An alternative view, only occasionally expressed, saw the difference between those who had tests and treatment and those who did not in psychological terms (see quote 4 in Table 4).

**Table 4:** Characteristics of those thought less likely to seek help

	
1	“…*there*’*s a general group of people*, *I think men are more likely to be in it than women*, *who don*’*t go to the doctor for many things that they ought to*.”
	“*I think with men it is a sort of slur on their manhood*”. [*FG3*]
2	“….*living on their own*, *they have no family nearby to keep chivvying them up*, *keep an eye on them*…*they may not be very mobile either*”. [*FG3*]
3	“…*it could be somebody can*’*t get there*”. [*FG1*]
	“…*it depends very much on the person*…*whether they*’*ve got an open mind*”. [*FG4*]

### Theme 2. Screening vs. the attribution of change to ageing

There was a widespread view that preventive care was important and helpful, and everyone should do it (quotes 1 and 2 in Table 5). Some people attributed all changes in eyesight to normal ageing, and therefore did not act on any changes they experienced (quotes 3 and 4 in Table 5).

**Table 5:** Preventive care vs. normal ageing

	
1	“ …. (*the optician*) *was the first person to detect that I had high blood pressure*, *because they can see so much*, *you know*, *through their machines*”. [*FG1*]
2	“*I believe they can detect heart conditions as well*”. [*FG1*]
3	.“….*there is a class of older person maybe on their own*, *who thinks that their deteriorating sight is part and parcel of getting older*”. [*FG3*]
4	“*Do you think sight loss is an unavoidable part of getting older*?” “*Oh I hope not*.” “*I think it is*”. [*FG4*]

### Theme 3. Professionalism vs. commercialisation

There was a strong sense of incongruence between trusting the skills and professionalism of opticians and distrusting the commercial motivation of the industry in which they worked. The focus groups were very vocal about the commercialism of optical services (quotes 1 to 7 in Table 6) but there were contrary views about the significance of commercialisation (see quotes 8 and 9 in Table 6).

**Table 6:** Professionalism and commercialization

	
1	“…*I*’*m sure the smaller*, *more dependable optician is far better for you in the long run*. *It might be dearer*, *but I*’*m sure you get a better service*.” [*Fg3*]
2	“*What they are really after is selling you glasses*, *I think*”. [*FG1*]
3	. “*They only call me back every year because they are looking for business*.” [*FG1*]
4	“*With vision*….*most people use what is really private medicine*, *because the optician is all about private medicine*”. [*FG2*]
5	“*They try and sell you these thin things now*, *you look like Dr*. *Spock in Star Trek*. *I mean*, *they*’*re not decent*.” [*FG2*]
6	“*You go to a dentist or you go to an optician and they tell you all the problems you*’*ve got because of that* (*gestures as if playing with money*)”. [*FG4*
7	“*But it*’*s very difficult*, *going to have your eyes tested*, *and they say your prescription has changed*, *to say* ‘*Well thank you very much*, *I*’*ll take my prescription somewhere else*’. *You feel you*’*re a captive audience there*”. [*FG5*]
8	“*I was quite favourably impressed with the commercial optician because they did seem to pick up the fact that there was something wrong at an early stage*”. [*FG1*]
9	“…*there is a consensus here that they are robbers*, *but they are not*.” [*FG1*]

**Figure 2:**
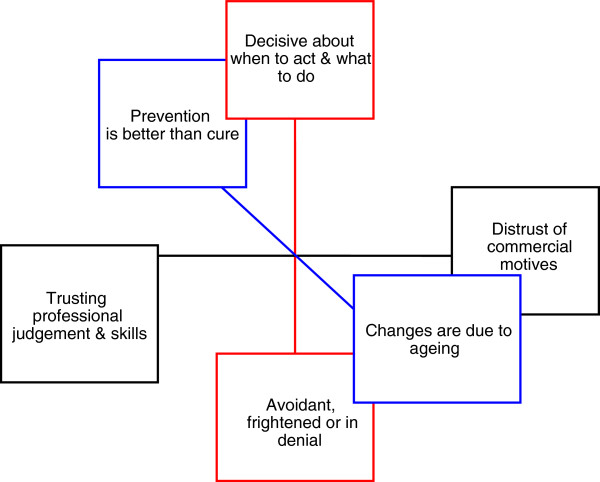
**Vision loss in later life: a model of factors influencing decisions &actions.**

We combined these polarised themes into a three dimensional model to illustrate why eye services are not used by some people. (See Figure 2).

## Discussion

### What this study shows

This qualitative study provides a complex model to explain why the experience of vision loss is not always acted on by older people. The model includes psychological attributes, costs to the individual and judgments about normal ageing. The three dimensions of the model could be used by practitioners to identify factors that keep individuals from seeking help and hence a group of higher–risk individuals, for case-finding for vision loss.

### Strengths and limitations of the study

The strengths of this study are that its theoretical framework and methods have been made explicit, and the sampling strategy was comprehensive to ensure generalisability of the conceptual analysis, and the number of participants was large. We have offered a detailed description of the fieldwork, and described the trustworthiness of our analysis through independent inspection of our data. The data analysis is clearly described and theoretically justified, and its reliability was ensured by having more than one researcher undertaking the analysis. We have described our search for contradictory perspectives. The limitations are that the sample was drawn from a single practice in suburban north London, which chose to continue their participation in a lengthy study of health risk appraisal, and that no different themes emerged from the individual interviews, compared with the focus groups.

### Comparison with the literature

A survey by the RNIB of 5,000 people aged 60 and over exploring the barriers to eye-testing, found that almost half (47%) do not have annual eye tests [22]. Sixty percent of respondents who had not had their eyes tested in the past two years said the main reason for not going was that they had not had a problem with their eyes, which is consistent with the difficulty with judgements about changing eyesight noted in this study. Eighteen percent said the cost of glasses was the main deterrent, rising to 21–26% in low income groups, which supports the views of participants in this study. Of those aged 80 and over, difficulties with access due to transport accounted for 25% of the respondents’ reports that they had not had their eyes checked. The importance given to psychological factors is consistent with the importance of self-efficacy in determining uptake of preventive health care in this study population [23].

### Implications for practice and research

Education and awareness of the medical and care community, in particular the role of general practitioners in promoting eye health, is a key area of action identified by the Eye Health Alliance [24]. Currently, visual assessment is only required under the Quality and Outcomes Framework (QOF), the annual incentive programme for disease management in general practice, for patients with diabetes. The characteristics of the high-risk group for undetected vision loss could be incorporated into a heuristic (rule of thumb) [25] to help practitioners to identify older patients whose vision could usefully be assessed. This potential heuristic needs development and testing in routine practice.

Another way in which this model can be used is to consider which axis is the most easily modified, so that early efforts to change the situation are more likely to be fruitful. For example, one way to reduce avoidant behaviour may be to reduce the perceived commercial interests of optometrists, by making their relationship with the NHS more visible.

## Conclusions

The reasons why older people may not seek help for deteriorating vision can be explained in a model in which psychological attributes, costs to the individual and judgments about normal ageing interact. Understanding this model may help clinical decision making.

## Competing interests

The authors declare that they have no competing interests.

## Authors’ contributions

KK developed the study design, collected and analysed data and drafted this paper, SI developed the study design, analysed data and contributed to this paper, SM collected and analysed data. All authors read and approved the final manuscript.

## Pre-publication history

The pre-publication history for this paper can be accessed here:

http://www.biomedcentral.com/1471-2296/14/99/prepub
